# Copper ion salts of arylthiotetrathiafulvalenes: synthesis, structure diversity and magnetic properties

**DOI:** 10.3762/bjoc.11.95

**Published:** 2015-05-20

**Authors:** Longfei Ma, Jibin Sun, Xiaofeng Lu, Shangxi Zhang, Hui Qi, Lei Liu, Yongliang Shao, Xiangfeng Shao

**Affiliations:** 1 State Key Laboratory of Applied Organic Chemistry, Lanzhou University, Tianshui Southern Road 222, Lanzhou 730000, Gansu Province, China

**Keywords:** antiferromagnetic interaction, arylthio-substituted tetrathiafulvalenes, charge-transfer, crystal structure, magnetic property

## Abstract

The combination of CuBr_2_ and arylthio-substituted tetrathiafulvalene derivatives (**1**–**7**) results in a series of charge-transfer (CT) complexes. Crystallographic studies indicate that the anions in the complexes, which are derived from CuBr_2_, show diverse configurations including linear [Cu(I)Br_2_]^–^, tetrahedral [Cu(II)Br_4_]^2–^, planar [Cu(II)_2_Br_6_]^2–^, and coexistence of planar [Cu(II)Br_4_]^2–^ and tetrahedral [Cu(II)Br_3_]^–^ ions. On the other hand, the TTFs show either radical cation or dication states that depend on their redox potentials. The central TTF framework on most of TTFs is nearly planar despite the charge on them, whereas the two dithiole rings on molecule **4** in complex **4**·CuBr_4_ are significantly twisted with a dihedral angle of 38.3°. The magnetic properties of the complexes were elucidated. The temperature-dependent magnetic susceptibility of complex **5**·Cu_2_Br_6_ shows the singlet–triplet transition with coupling constant *J* = −248 K, and that of **3**·(CuBr_4_)_0.5_·CuBr_3_·THF shows the abrupt change at 270 K caused by the modulation of intermolecular interactions. The thermo variation of magnetic susceptibility for the other complexes follows the Curie–Weiss law, indicating the weak antiferromagnetic interaction at low temperature.

## Introduction

Since firstly synthesized in 1970s [1], tetrathiafulvalene (TTF) and its derivatives have been intensively studied to explore functional organic materials [2]. Inspired by the discovery of highly conducting charge-transfer (CT) complex TTF·TCNQ [3] and the first organic superconductor (TMTSF)_2_X [4], the chemical modifications on TTF are traditionally aimed at the creation of organic conductors with various electronic ground states [5–10]. It has been well-defined that a subtle modification of TTF would result in a significant effect on the properties of their complexes [5–10]. For example, the complexes of EDO-TTF (4,5-ethylenedioxy-TTF) [11–15] and MeEDO-TTF (4,5-ethylenedioxy-4’-methyl-TTF) [16–19] show the distinct difference on electrical transport properties. Meanwhile, the modification on TTF, particularly introducing aromatic substituents onto the TTF core, is one of the key strategies to explore functional molecular materials. The resulting TTFs have been employed as electrochemically active units in supramolecular systems and/or molecular devices, which has been summarized in many reviews [20–32]. However, the incorporation of aryl groups onto the TTF core through sulfur bridges, which resulted in arylthio-substituted TTFs (denoted as Ar-S-TTF), has been scarcely reported due to synthetic difficulties [33–36]. Recently, we have disclosed a facile approach toward Ar-S-TTFs [37]. Crystallographic investigations indicate that Ar-S-TTFs show various molecular geometries and packing structures depending on the nature of the peripheral aryls [38–39].

**Scheme 1:**
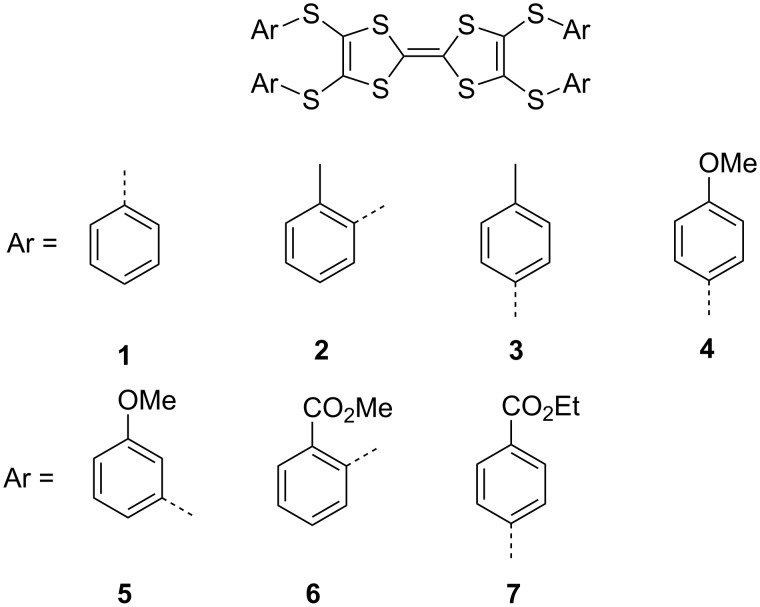
Chemical structures of arylthio-substituted tetrathiafulvalenes (**1**–**7**).

The TTF-based conducting materials are mainly produced as radical cation salts by electrochemical oxidation and CT complexes by chemical oxidation with electron acceptors [5–6]. Most Ar-S-TTFs possess redox potentials higher than that of bis(ethylenedithio)-TTF (BEDT-TTF) [33–39]. Consequently, the complexes of Ar-S-TTFs with electron acceptors such as fullerenes [40–41] and TCNQ [42] show a neutral ground state. However, Ar-S-TTFs can be chemically oxidized by strong electron acceptors such as F_4_TCNQ [42] and Keggin-type phosphomolybdic acid [43] to form CT complexes. In comparison with fullerenes and TCNQ, the inorganic salt CuX_2_ (X = Cl, Br) is a strong oxidant and has been used to oxidize the TTFs to form organic conductors with diverse electronic ground states [44–54]. Herein, we report the synthesis, structure, and magnetic properties of the complexes of Ar-S-TTFs (**1**–**7**, Scheme 1) with CuBr_2_. These complexes show diverse structures and properties related to the oxidation state as well as the molecular geometries of TTFs.

## Results and Discussion

### Synthesis

The donor molecules (**1**–**7**, Scheme 1) were synthesized according to our previous report [37–38], and their electrochemical activities as well as the crystal structures have been fully elucidated [38–39]. Since the redox potentials of TTFs are very important in the formation of complexes, particularly on the charge-transfer degree, the first (*E*_1/2_^1^) and the second (*E*_1/2_^2^) redox potentials of **1**–**7** are summarized in Table 1. As reported in the following section, TTFs **1**–**5** have the *E*_1/2_^2^ < 0.90 V and form the dicationic salts by reaction with CuBr_2_. On the contrary, the *E*_1/2_^2^ values of **6** and **7** are higher than 0.90 V, and these two donor molecules form the radical cation salts by reaction with CuBr_2_.

**Table 1:** Electrochemical data of TTFs in this report.^a^

	**1**	**2**	**3**	**4**	**5**	**6**	**7**

*E*_1/2_^1^ [V]	0.56	0.52	0.51	0.48	0.56	0.62	0.66
*E*_1/2_^2^ [V]	0.89	0.85	0.85	0.83	0.88	0.95	0.96
*∆E* [V]^b^	0.33	0.33	0.34	0.35	0.32	0.33	0.30

^a^See reference [38], and the redox potentials are recorded vs SCE; ^b^*∆E = E*_1/2_^2^ − *E*_1/2_^1^_._

The reaction of **1**–**7** with CuBr_2_ was performed in the mixed solvent of tetrahydrofuran–acetonitrile (THF–CH_3_CN; v/v, 1:1) at room temperature. In the low concentration (<10^−4^ mol L^−1^), a dark green solution was formed, indicating the oxidation of **1**–**7** by CuBr_2_. When the concentration of the reaction system was increased to higher than 10^−3^ mol L^−1^, TTFs **1**–**7** afforded the ionic salts showing the same phase as those of the corresponding single crystalline ones. The single crystalline salts were obtained by a conventional two-phase diffusion method. In a typical procedure, the CuBr_2_ solution in CH_3_CN and the solution of TTFs in THF were placed in two different chambers of an H-shape cell, respectively. After several weeks, black single crystalline salts were formed. The compositions of the salts were determined by X-ray single crystal diffraction analyses, as summarized in Table 2.

**Table 2:** Preparation, composition, yield, and appearance of the salts.^a^

TTFs		salts^b^		
	amount	composition	amount (yield)	appearance^c^
	
**1**	19 mg (0.03 mmol)	**1**·CuBr_4_	24 mg (80%)	black needle
**2**	28 mg (0.04 mmol)	**2**·CuBr_4_	39 mg (91%)	black needle
**3**	28 mg (0.04 mmol)	**3**·(CuBr_4_)_0.5_·CuBr_3_·THF	46 mg (92%)	black block
**4**	23 mg (0.03 mmol)	**4**·CuBr_4_	31 mg (91%)	black needle
**5**	23 mg (0.03 mmol)	**5**·Cu_2_Br_6_	33 mg (80%)	black block
**6**	26 mg (0.03 mmol)	**6**·CuBr_2_·CH_3_CN	26 mg (74%)	black block
**7**	28 mg (0.03 mmol)	**7**·CuBr_2_	16 mg (47%)	black cuboid

^a^TTFs were dissolved in 4 mL of THF, and CuBr_2_ (100 mg, dissolved in 4 mL of CH_3_CN) was applied in the synthesis. ^b^The compositions were determined by X-ray single crystal diffraction analyses. ^c^See the photographs of the crystals in Figure S1 in Supporting Information File 1.

### Crystal structure

**Figure 1:**
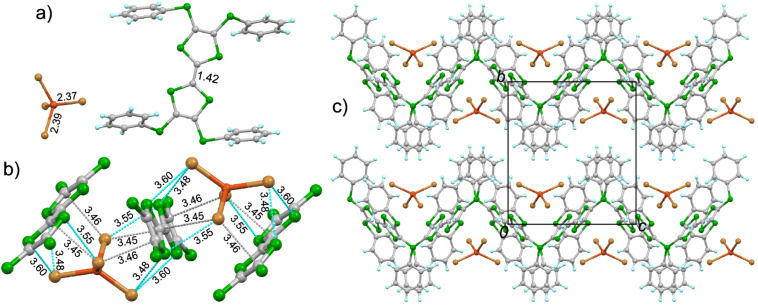
Crystal structure of **1**·CuBr_4_: a) unit cell contents with the typical bond lengths shown (in Å); b) interactions between the [Cu(II)Br_4_]^2−^ ion and the central TTF core of **1**, where the cyan and grey dashed lines represent Br···S and Br···C contacts (Å), respectively; c) packing structure viewed along the *a*-axis.

**Figure 2:**
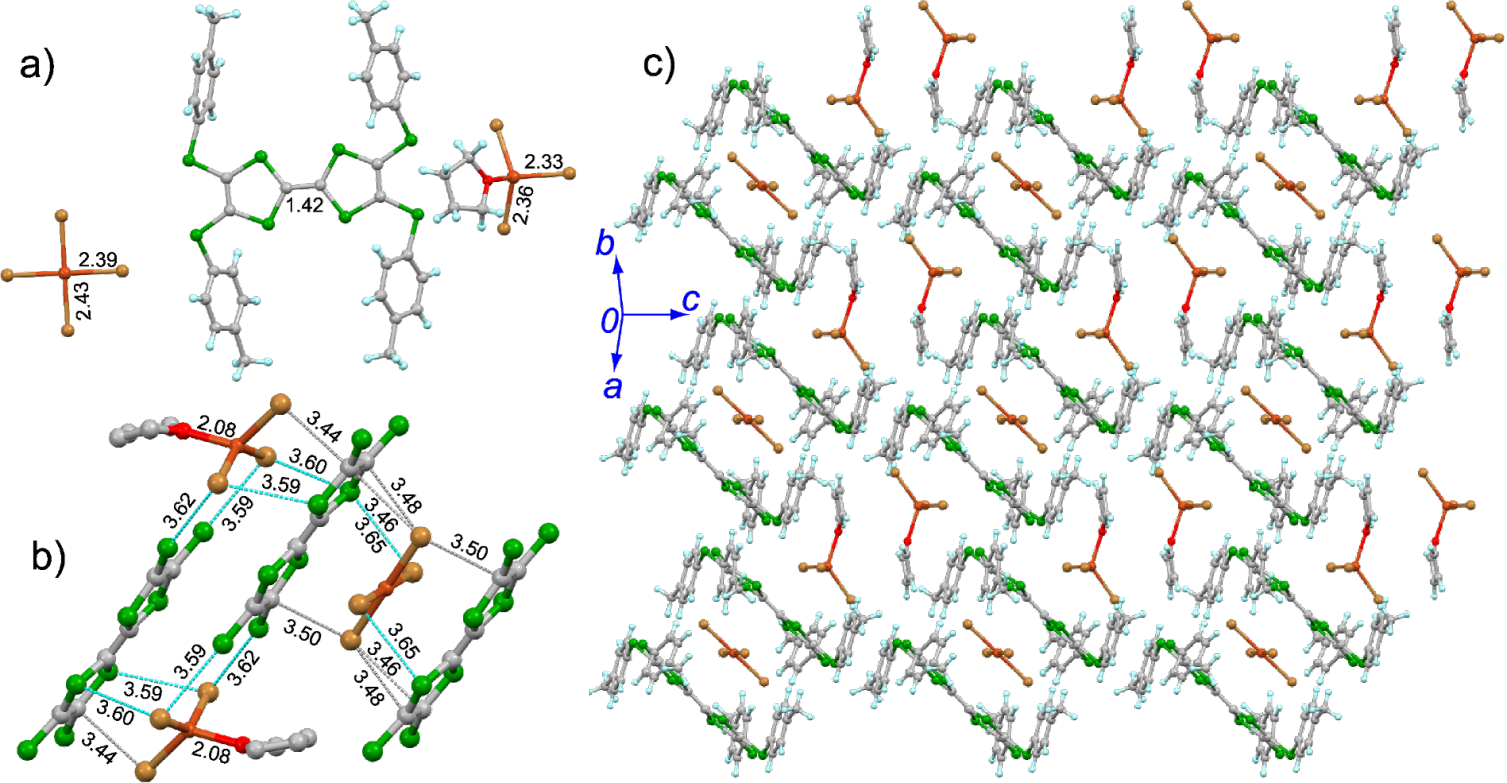
Crystal structure of **3**·(CuBr_4_)_0.5_·CuBr_3_·THF: a) unit cell contents with the typical bond lengths shown (Å), and the structure of planar [Cu(II)Br_4_]^2−^ ion is generated by symmetry operation (2-*x*, 2-*y*,1-*z*); b) interaction between the anions and the central TTF core of **3**, where cyan and grey dashed lines represent Br···S and Br···C contacts (Å), respectively; c) packing structure.

**Figure 3:**
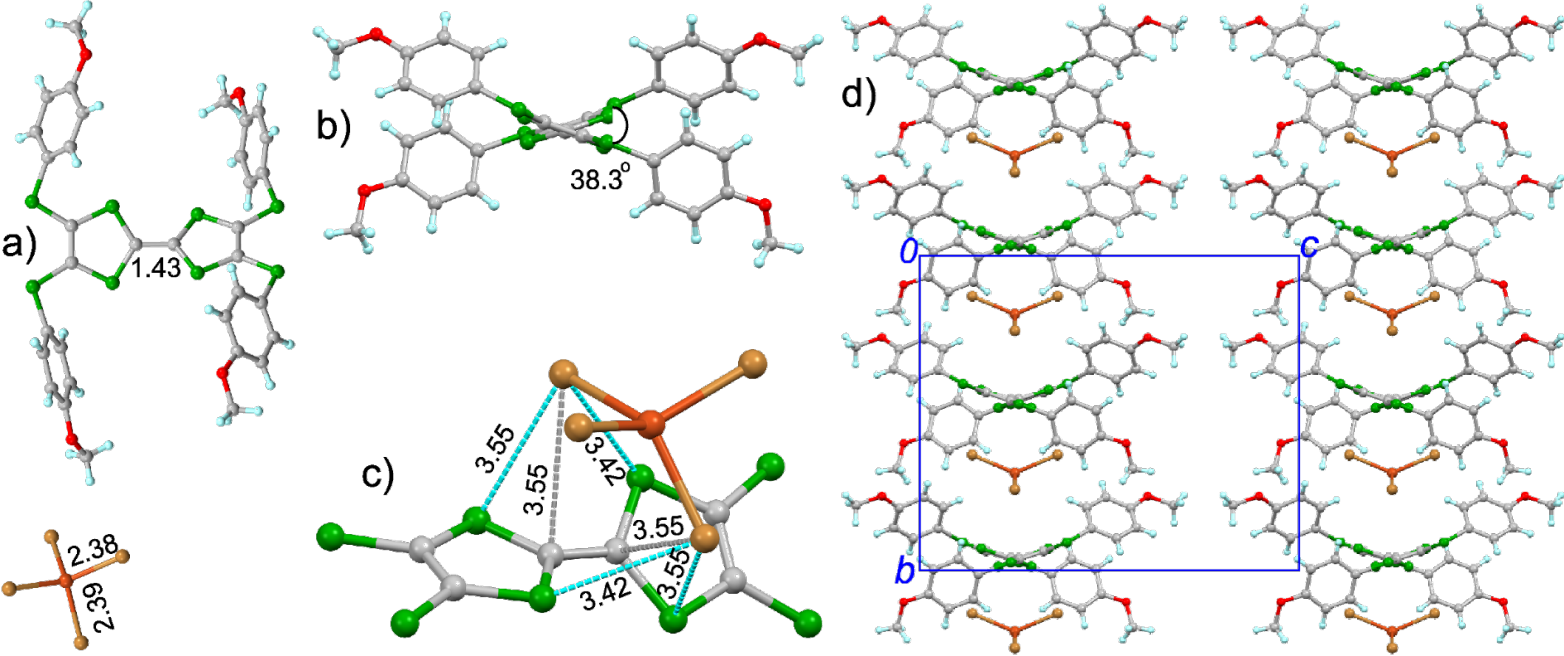
Crystal structure of **4**·CuBr_4_: a) unit cell contents with the typical bond lengths shown (Å); b) view of molecule **4** along the central C=C bond of the TTF core, and the dihedral angle between two C_3_S_2_ rings is shown; c) interaction between the [Cu(II)Br_4_]^2−^ ion and the central TTF core of **4**, where cyan and grey dashed lines represent Br···S and Br···C contacts (Å), respectively; d) packing structure viewed along the *a*-axis.

**Figure 4:**
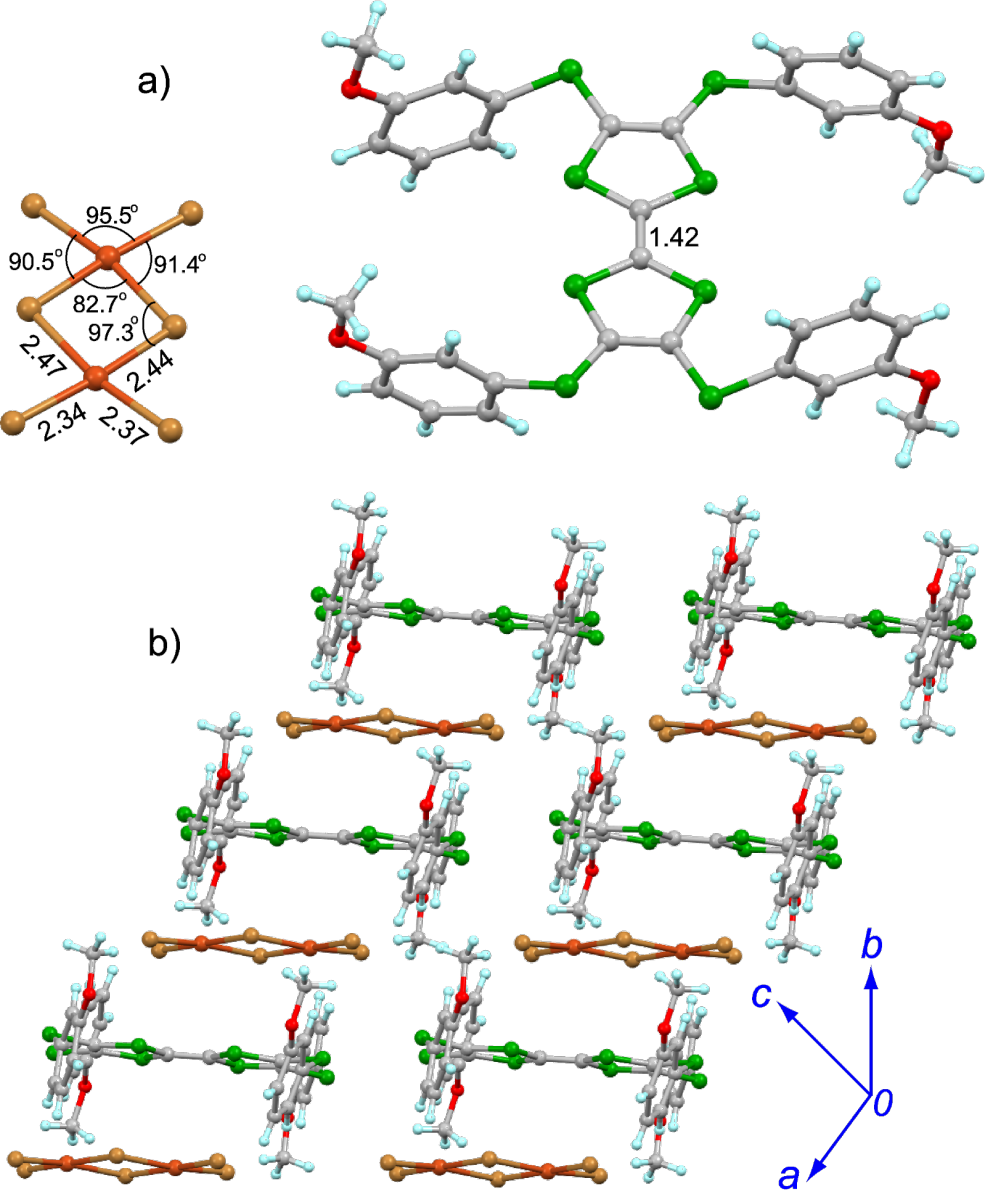
Crystal structure of **5**·Cu_2_Br_6_: a) unit cell contents with the numeric data indicate the angles and bond lengths (Å); b) packing structure.

**Figure 5:**
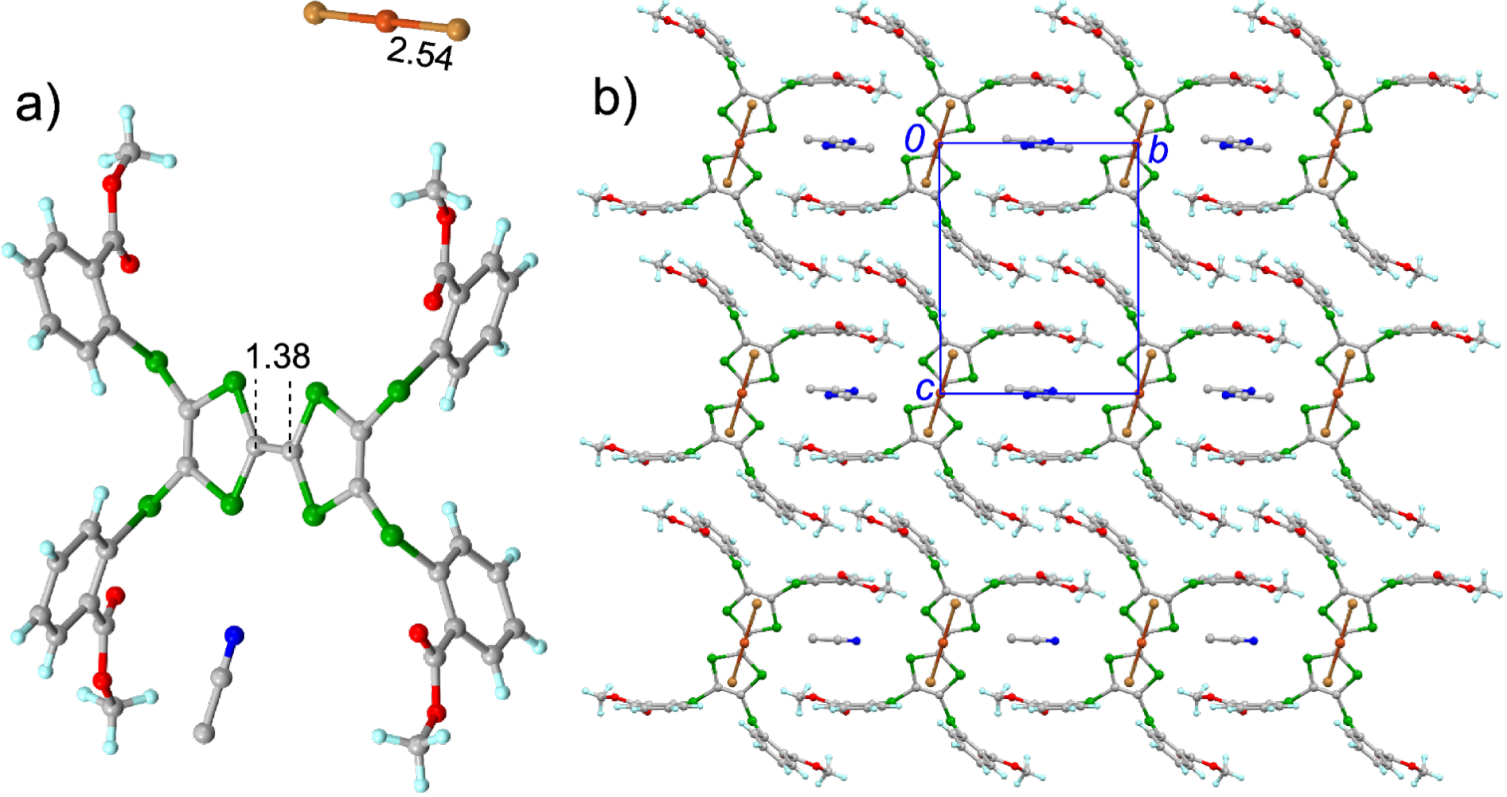
Crystal structure of **6**·CuBr_2_·CH_3_CN: a) unit cell contents with the typical bond lengths shown (Å); b) packing structure viewed along the crystallographic *a*-axis.

The single crystals for all of the present salts were suitable for the X-ray single crystal diffraction analyses. Herein, we report the crystal structures of the typical salts (Figures 1–5), and those of the others are supplied in Supporting Information File 1. As mentioned above, the molecular geometries of Ar-S-TTF are sensitive to the environmental variations, especially the guest components are included in their solid-state matrix. Besides, the bond lengths and the conformation of the central TTF core are sensitive to the charge variation. The charge on TTFs can be estimated according to an empirical formula suggested by Day et al. [55], that is δ = (*b* + *c*) − (*a* + *d*). The calculated δ values and the conformation of TTFs **1**–**7** in neutral state and salts are summarized in Table 3. These results indicate that **1**–**5** have the charge of +2, whereas **6** and **7** are radical cations. The central TTF cores on the neutral TTFs show various conformations including chair, planar, and boat conformations. However, the central TTF cores of TTFs in the present salts are planar except that of **4**, where the two dithiole rings are twisted with a dihedral angle of 38.3°. In the following, we will discuss the crystal structures of these salts, including the molecular geometry of TTFs, the structure of anions, and the packing motifs.

**Table 3:** Selected bond lengths, calculated charge, and conformations of TTFs.

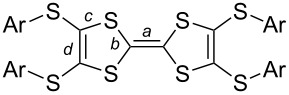								

		*a* [Å]	*b* [Å]	*c* [Å]	*d* [Å]	δ [Å]	charge	conformation

**1**	neutral^a^	1.325	1.740	1.737	1.325	0.827	0	chair
	complex	1.421	1.689	1.709	1.367	0.608	+2	planar

**2**	neutral^a^	1.329	1.764	1.750	1.351	0.834	0	planar
	complex	1.422	1.682	1.708	1.364	0.604	+2	planar

**3**	neutral^a^	1.340	1.757	1.756	1.333	0.840	0	boat
	complex	1.418	1.693	1.704	1.375	0.604	+2	planar

**4**	neutral^a^	1.342	1.761	1.754	1.339	0.834	0	chair
	complex	1.428	1.685	1.700	1.379	0.578	+2	twist

**5**	neutral	–	–	–	–	–	–	–
	complex	1.429	1.693	1.710	1.379	0.595	+2	planar

**6**	neutral^a^	1.336	1.757	1.756	1.345	0.832	0	chair
	complex	1.382	1.716	1.746	1.336	0.744	+1	planar

**7**	neutral^a^	1.34	1.76	1.76	1.34	0.84	0	planar
	complex	1.39	1.71	1.74	1.35	0.71	+1	planar

^a^See reference [38].

**1**·CuBr_4_ crystallizes in the orthorhombic *Pbcn* space group with half of molecule **1** and half of CuBr_4_ crystallographically unique (Figure 1a). The central TTF core on **1** is nearly planar, which is different from the chair conformation in the neutral state. Moreover, the spatial alignment of peripheral phenyls is modulated (see Figure S2 in Supporting Information File 1). The calculated δ value of **1** is 0.608 in the salt, indicating it has the charge of +2 according to the criteria proposed by Day [55]. The inorganic component CuBr_4_ takes the slightly distorted tetrahedral geometry. The Cu–Br bond lengths are 2.37 and 2.39 Å, which are close to a typical Cu(II)–Br bond length [51–53,56–58]. Thus, the CuBr_4_ component should have the charge of −2, consistent with the dicationic state of **1**. A [CuBr_4_]^2−^ ion is encapsulated by two donor molecules, and there are multiple Br···S (3.48–3.60 Å) and Br···C (3.45–3.46 Å) close contacts [59] between the [CuBr_4_]^2−^ ion and the central TTF core of **1** (Figure 1b). Donor molecules form a zig-zag chain alignment along the *c*-axis (Figure 1c), and the [CuBr_4_]^2−^ ion locate at the cavity formed by **1**. The spin exchange interaction between Cu(II) on [CuBr_4_]^2−^ ions would take place as mediated by the π-orbitals of **1**. The crystal structure of **2**·CuBr_4_ is reminiscent to that of **1**·CuBr_4_ as shown in Figure S3 and Figure S4 in Supporting Information File 1.

The crystal structure of **3**·(CuBr_4_)_0.5_·CuBr_3_·THF at room temperature is shown in Figure 2. This salt crystallizes in the triclinic *P*−1 space group, and the asymmetric unit contains one molecule **3**, half of CuBr_4_, one CuBr_3_, and one THF. The central TTF core of **3** takes the planar conformation similar to its neutral state, whereas the spatial alignment of the 4-tolyl groups is altered (Figure S6 in Supporting Information File 1). The calculated δ value of **3** in the salt is 0.604, indicating that **3** is oxidized to the dication form. The inorganic component CuBr_4_ has a planar conformation with a Cu–Br bond length of 2.39 and 2.43 Å (Figure 1a), thus it should be dianionic. On the other hand, the oxygen atom on THF coordinates to the Cu atom on CuBr_3_ with a Cu–O bond length of 2.08 Å, consequently CuBr_3_ takes a distorted tetrahedral conformation. The Cu–Br bond length in CuBr_3_ is 2.33–2.36 Å, indicating that the Cu atom in CuBr_3_ should be Cu(II). A [CuBr_4_]^2−^ ion is sandwiched by two donor molecules through the Br···S (3.65 Å) and Br···C contacts (3.46–3.50 Å), thus, a D–A–D type trimer of [**3**–CuBr_4_–**3**] is formed as shown in Figure 2b. The neighboring D–A–D trimers shift along the longitudinal axis of **3**, thus form a voidage to accommodate one [CuBr_3_·THF] as shown in Figure 2c. There are Br···S (3.59–3.62 Å) and Br···C contacts (3.44 Å) between the [CuBr_3_]^−^ ion and molecule **3** in the D–A–D trimer. Consequently, the spin interaction between Cu(II) is expected, which would be mediated through the π-orbitals of **3**. The packing structure of this salt at low temperature (173 K) is very similar to that at room temperature, whereas the intermolecular interactions between the organic and inorganic components are strengthened, particularly for those between [CuBr_3_·THF] and D–A–D timers (Figure S5 in Supporting Information File 1), which would result in the significant effect on the magnetic property as discussed in the following section.

**4**·CuBr_4_ crystallizes in the orthorhombic *Pccn* space group with half of molecule **4** and half of CuBr_4_ crystallographically independent (Figure 3a). The calculated δ value of **4** in the salt is 0.578, indicating that the charge on **4** should be +2. The two dithiole rings of molecule **4** are significantly twisted with a dihedral angle of 38.3° as shown in Figure 3b. The CuBr_4_ component shows the tetrahedral conformation with a Cu–Br bond length of 2.38 and 2.39 Å, thus CuBr_4_ is a dianion. The [CuBr_4_]^2−^ ion locates above the donor molecule, and there are Br···S (3.42–3.55 Å) and Br···C contacts (3.55 Å) between the [CuBr_4_]^2−^ ion and **4** as shown in Figure 3c. Molecule **4** and the [CuBr_4_]^2−^ ion form the mixed aggregation along the *b*-axis (Figure 3d). Although there is no interaction between the neighbouring donor molecules in the *bc*-plane, the S···S contacts (3.22 Å) are observed between the molecules of **4** along the *a*-axis direction, which would result in the spin exchange interaction between Cu(II).

**5**·Cu_2_Br_6_ crystallizes in the triclinic *P*−1 space group with half of molecule **5** and half of Cu_2_Br_6_ crystallographically independent (Figure 4a). The central TTF core of **5** adopts the planar conformation, and the δ value of **5** in the salt is 0.595, indicating that **5** is oxidized to the dication form. As for the inorganic component Cu_2_Br_6_, two Cu atoms are connected by two bromine bridges (Br–Cu bond length: 2.47 Å) to form a quasi-planar dianion [Cu_2_Br_6_]^2−^. Thus, the spin exchange interaction between these two Cu(II) would be significant, as discussed in the following section. Molecule **5** and the [Cu_2_Br_6_]^2−^ ion form the mixed aggregation along the *b*-axis as shown in Figure 4b. There is no atomic close contact between the organic and inorganic components in a stacking column, whereas one S···S contact (3.57 Å) is observed between the neighbouring molecules of **5** along their longitudinal axis.

**6**·CuBr_2_·CH_3_CN crystallizes in the triclinic *P*−1 space group, and the asymmetric unit contains half of molecule **6**, half of CuBr_2_, and half of a CH_3_CN solvent molecule (Figure 5a). The central TTF core of **6** has a pseudo-planar conformation, and the calculated δ value of **6** in the salt is 0.744, indicating that **6** is in the radical cation form. The inorganic component CuBr_2_ is linear, and the Cu–Br bond length is 2.54 Å, which is close to that of a typical Cu(I)–Br bond [51–53,56–58], indicating that CuBr_2_ has the charge of −1. The organic and inorganic components form the mixed stacks along the *a*-axis as shown in Figure 5b. Moreover, the peripheral aryl groups form the cavity to accommodate a CH_3_CN solvent molecule, thus a supramolecular framework is formed in this salt. In the salt of **7** with CuBr_2_, molecule **7** is also oxidized to the radical cation form and the counter anion is [CuBr_2_]^−^ as shown in Supporting Information File 1 (Figure S11).

### Magnetic properties

**Figure 6:**
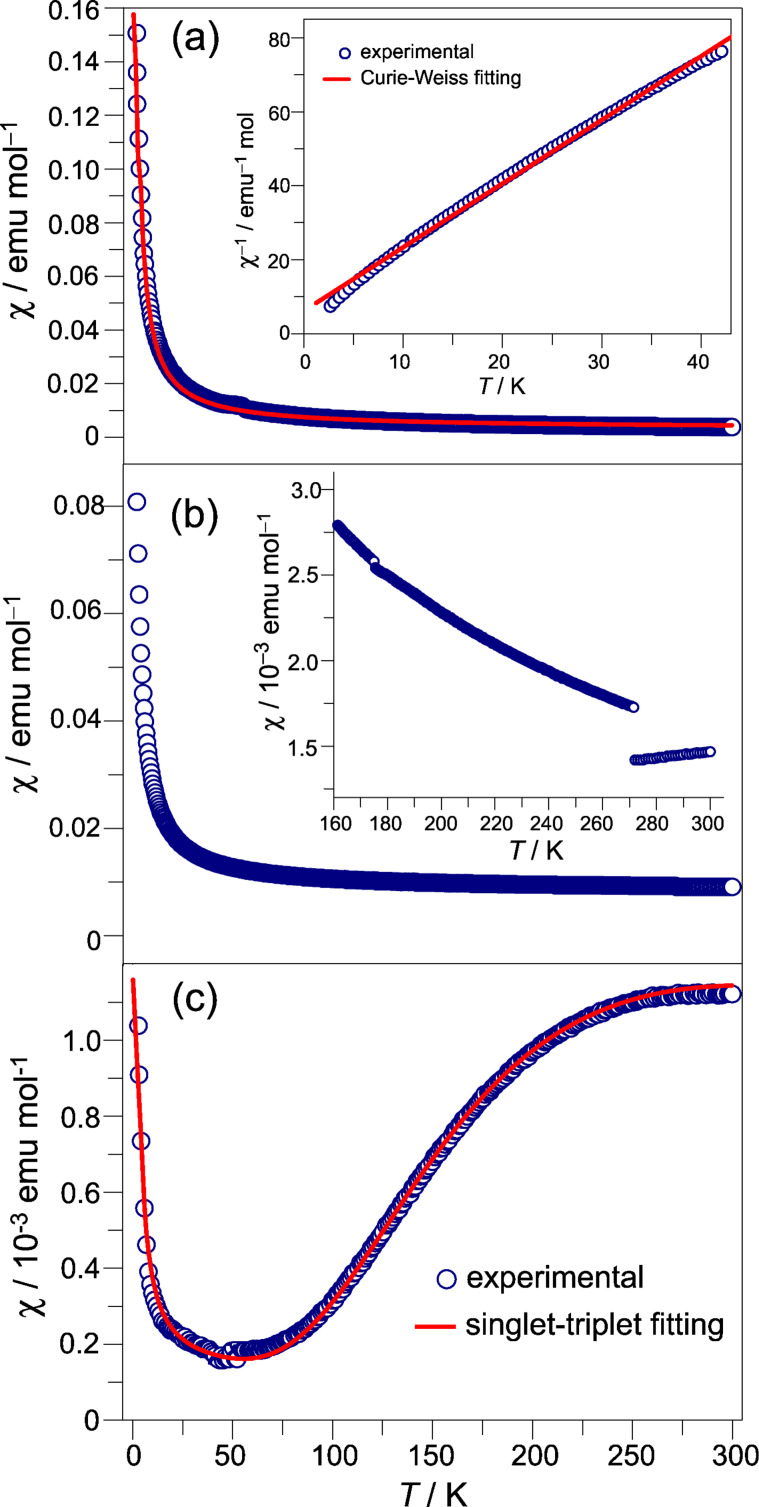
Temperature dependent magnetic susceptibility for the typical salts: a) **1**·CuBr_4_ with the insert panel depicting the Curie–Weiss fitting (red solid line) in low temperature region; b) **3**·(CuBr_4_)_0.5_·CuBr_3_·THF with the insert panel depicting the magnetic susceptibility at high temperature region; c) **5**·Cu_2_Br_6_ with the red solid line depicting the singlet-triplet fitting.

The temperature-dependent magnetic susceptibilities of the salts were measured on the polycrystalline samples. In the salts of **1**–**5**, the spin susceptibility comes from Cu(II) (*S* = 1/2), because the TTFs in these salts are oxidized to the dication form and the inorganic components contain Cu(II). On the other hand, spin susceptibility on the salts of **6** and **7** originates from the radical cation, as the inorganic components in these salts contain Cu(I). Figure 6 depicts the temperature-dependent magnetic susceptibilities of the representative salts.

**1**·CuBr_4_, **2**·CuBr_4_, **4**·CuBr_4_, and **7**·CuBr_2_ show the similar magnetic properties. The temperature dependence of the magnetic susceptibility follows the Curie–Weiss law, and the spins in these salts show the antiferromagnetic interaction at low temperature. The antiferromagnetic interactions of Cu(II) in **1**·CuBr_4_, **2**·CuBr_4_, and **4**·CuBr_4_ arise from the *d*–π–*d* pathway, as discussed in the crystal structure section. On the other hand, the antiferromagnetic interaction of radical cations in **7**·CuBr_2_ could be due to the π–π interactions, because the neighbouring donor molecules have a S···S contact (3.30 Å) along the *a*-axis. Figure 6a shows the magnetic susceptibility of **1**·CuBr_4_ by varying temperature, and the best-fitting parameters for this salt are *C* = 0.382 emu K mol^−1^ and θ = −5.4 K.

In the case of **3**·(CuBr_4_)_0.5_·CuBr_3_·THF, the temperature dependence of magnetic susceptibility shows the monotonic decrement upon cooling in the temperature range of 300–270 K. Furthermore, an abrupt jump of the magnetic susceptibility is observed at 270 K (see Figure 6b). This abrupt jump could be attributed to the variation of intermolecular interactions as discussed in the crystal structure section. Below 270 K, the temperature dependence of magnetic susceptibility follows the Curie–Weiss law with *C* = 0.379 emu K mol^−1^ and θ = −4.6 K.

As mentioned in the crystal structure section, two Cu(II) atoms in **5**·Cu_2_Br_6_ are connected by two bromine bridges, which result in the strong spin interaction between Cu(II) atoms. The temperature-dependent magnetic susceptibility of **5**·Cu_2_Br_6_ is shown in Figure 6c, which can be well-fitted by the singlet–triplet model [60]. The best-fitting parameters are: *J* = −243 K which is consistent with the significant magnetic susceptibility dropping at 245 K, *f* = 0.993, and *A* = 3.21 × 10^−4^ emu mol^−1^. The latter two terms reflect the non-zero magnetic susceptibility originated from the crystal defects (the Curie term) and the residue paramagnetic impurities.


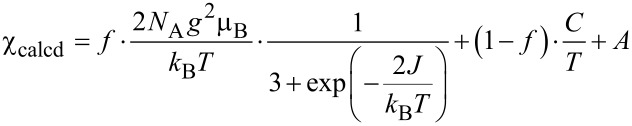


## Conclusion

We have reported the synthesis, structures, and magnetic properties of the copper ion salts of Ar-S-TTFs **1**–**7**. The present salts show a wide variety of solid state structures and magnetic properties. The charge on TTFs in the salts depends on their second redox potentials (*E*_1/2_^2^): *E*_1/2_^2^ > 0.90 V, radical cation; *E*_1/2_^2^ < 0.90 V, dication. Except compound **4**, which has the twisted central TTF core in the dicationic salt **4**·CuBr_4_, the central TTF frameworks of these TTFs are nearly planar despite the charge on them. On the other hand, the anions in the salts show various configurations including the linear [Cu(I)Br_2_]^−^ ion, the tetrahedral [Cu(II)Br_4_]^2−^ ion, the planar [Cu(II)_2_Br_6_]^2−^ ion, the planar [Cu(II)Br_4_]^2−^ ion, and the distorted tetrahedral [Cu(II)Br_3_·THF]^−^ ion. As a result of diverse geometries for both donor molecules and counter anions, the present salts show various packing structures, which results in a different spin-exchange interaction pathway as proved by their magnetic properties.

## Experimental

Cupric bromide (CuBr_2_) was purchased from Shanghai Xinbao Fine Chemical Factory (Shanghai, China). Tetrahydrofuran (THF) and acetonitrile (CH_3_CN) were distilled over CaH_2_ and stored under N_2_ atomsphere. Compounds **1**–**7** were synthesized by following our previous reports [37–38].

The electrochemical properties of **1**–**7** were recorded on a RST 5000 electrochemical workstation at a scan rate of 50 mV s^−1^, with glassy carbon discs as the working electrode, Pt wire as the counter electrode, and a SCE electrode as the reference electrode. The concentration was 5 × 10^−4^ mol L^−1^ in CH_2_Cl_2_, and the supporting electrolyte was (*n*-Bu)_4_N·PF_6_ (0.1 mol L^−1^). The measurement was performed at 20 °C after bubbling the solution with N_2_ gas for 15 min.

The X-ray diffraction measurement was carried out on SuperNova (Agilent) type diffractometer. The crystal structure was solved by a direct method SIR2004 [61] and refined by a full-matrix least-squares method on *F*^2^ by means of SHELXL-97 [62]. The X-ray powder diffraction (XRPD) pattern was recorded on X’Pert PRO (PANalytical). The temperature dependence of the magnetic susceptibility was measured on a SQUID magnetometer of Quantum Design MPMS-XL applying a magnetic field of 1 kOe. The data were corrected for core diamagnetism estimated from the sum of the Pascal constants [63].

## Supporting Information

File 1 Selected crystallographic data, crystal structures of **2**·CuBr_4_ and **7**·CuBr_2_, and variations of molecular geometries of TTFs at different oxidation states.

File 2 Crystallographic data files of compounds **1–7**. These data have been deposited to the Cambridge Crystallographic Data Centre (CCDC) with the registered numbers 1046215–1046222.
