# Supplementary material for: Copper ion salts of arylthiotetrathiafulvalenes: synthesis, structure diversity and magnetic properties
Source: Beilstein J Org Chem. 2015 May 20;11:850–9. doi: 10.3762/bjoc.11.95 (PMC4464265; doi:10.3762/bjoc.11.95)
Supplement: File 1 — Selected crystallographic data, crystal structures of 2·CuBr4 and 7·CuBr2, and variations of molecular geometries of TTFs at different oxidation states. [file Beilstein_J_Org_Chem-11-850-s001.pdf]

Supporting Information

for

Copper ion salts of arylthiotetrathiafulvalenes: synthesis, structure diversity and magnetic properties

Longfei Ma, Jibin Sun, Xiaofeng Lu, Shangxi Zhang, Hui Qi, Lei Liu, Yongliang Shao and Xiangfeng Shao*

Address: State Key Laboratory of Applied Organic Chemistry, Lanzhou University, Tianshui Southern Road 222, Lanzhou 730000, Gansu Province, China

Email: Xiangfeng Shao - shaoxf@lzu.edu.cn

*Corresponding author

Selected crystallographic data, crystal structures of 2·$CuBr_4$ and 7·$CuBr_2$, and variations of molecular geometries of TTFs at different oxidation states

Materials and general methods

$CuBr_2$ was purchased from Shanghai Xinbao Fine Chemical Factory (Shanghai, China). tetrahydrofuran(THF) and acetonitrile (CH_3CN) were distilled over CaH_2 and stored under N_2 atomsphere. **1**–**7** were synthesized by following our previous report [1].

The electrochemical properties of **1**–**7** were recorded on a RST 5000 electrochemical workstation at a scan rate of 50 mV s^{-1}, with glassy carbon discs as the working electrode, Pt wire as the counter electrode, and SCE electrode as the reference electrode. The concentration was 5×10^{-4} mol L^{-1} in CH_2Cl_2, and the supporting electrolyte was $(n\text{-Bu})_4N{\cdot}PF_6$ (0.1 mol L^{-1}). The measurement was performed at 20 ^{o}C after bubbling the solution with N_2 gas for 15 min.

The X-ray diffraction measurement was carried out on SuperNova (Agilent) type diffractometer. The crystal structure was solved by solved by a direct method SIR2004 [2] and refined by full-matrix least-squares method on F^2 by means of SHELXL-97 [3]. The X-Ray powder diffraction (XRPD) pattern was recorded on X'Pert PRO (PANalytical). The temperature dependence of the magnetic susceptibility was measured on a SQUID magnetometer of Quantum Design MPMS-XL applying a magnetic field of 1000 Oe. The data were corrected for core diamagnetism estimated from the sum of the Pascal constants [4].

References

[1] a) Sun, J.; Lu, X.; Shao, J.; Li, X.; Zhang, S.; Wang, B.; Zhao, J.; Shao, Y.; Fang, R.; Wang, Z.; Yu, W.; Shao, X. *Chem. Eur. J.* **2013**, *19*, 12517; doi: 10.1002/chem.201301819 b) Sun, J.; Lu, X.; Shao, J.; Cui, Z.; Shao, Y.; Jiang, G.; Yu, W.; Shao, X. *RSC Adv.* **2013**, *3*, 10193. doi: 10.1039/C3RA41349G

[2] Burla, M. C.; Caliandro, R.; Camalli, M.; Carrozzini, B.; Cascarano, G. L.; de Caro, L.; Giacovazzo, C.; Polidori, G.; Spagna, R. *J. Appl. Cryst.* **2005,** *38*, 381–388. doi: 10.1107/S0021889 80403225X

[3] Sheldrick, G. M. *SHELXL-97, A Program for Crystal Structure Refinement.* University of Göttingen, Göttingen, Germany, **1997**.

[4] Gupta, R. R.; *Landolt-Bornstein, New Sreies II* (Eds: K, -H. Hellwege, A. M. Hellwege), Springer Verlag, Berlin, **1986**, vol. 16 (Diamagnetism Susceptibility)

Table S1: Selected crystallographic data for **1**·$CuBr_4$, **2**·$CuBr_4$, and **3**·$(CuBr_4)_{0.5}$·$CuBr_3$·THF.

	**1**·$CuBr_4$	**2**·$CuBr_4$	**3**·$(CuBr_4)_{0.5}$·$CuBr_3$·THF	
CCDC number	1046215	1046216	1046217	1046218
Empirical formula	$C_{30}H_{20}S_8CuBr_4$	$C_{34}H_{28}S_8CuBr_4$	$C_{76}H_{72}O_2S_{16}Cu_3Br_{10}$	$C_{76}H_{72}O_2S_{16}Cu_3Br_{10}$
Formula weight	1020.12	1076.22	2520.02	2520.02
Temperature / K	293(2)	293(2)	173(2)	291(2)
λ / Å	1.54184	0.71073	0.71073	0.71073
Crystal size / mm^3	0.31×0.05×0.04	0.21×0.02×0.02	0.22×0.07×0.07	0.22×0.07×0.07
Crystal system	Orthorhombic	Monoclinic	Triclinic	Triclinic
space group	*P* bcn	*I*2/a	*P*-1	*P*-1
a / Å	21.0945(5)	13.9096(7)	13.0164(6)	13.1368(5)
b / Å	13.8077(7)	14.1046(7)	13.3435(6)	13.4389(5)
c / Å	12.4172(2)	21.0770(10)	14.5787(7)	14.8346(5)
α / °	90.00	90.00	102.051(6)	101.664(3)
β / °	90.00	107.985(6)	92.764(6)	93.457(3)
γ / °	90.00	90.00	116.503(7)	116.863(4)
V / $Å^3$	3616.7(2)	3933.0(3)	2194.51(18)	2253.37(14)
Z	4	4	1	1
d_{calc} / g·cm^{-3}	1.873	1.818	1.907	1.857
μ / mm^{-1}	10.522	5.069	5.698	5.549
$2\theta_{max}$ / °	145.34	57.34	57.74	57.82
Data / restraints / parameters	3582/0/195	4302/0/213	10203/0/488	10331/0/488
GooF	1.045	0.974	1.035	1.011
R [I>2σ(I)]	0.0458	0.0494	0.0532	0.0591
wR_2	0.1168	0.0831	0.1133	0.1228

Table S2: Selected crystallographic data for **4**·$CuBr_4$, **5**·Cu_2Br_6, **6**·$CuBr_2$·CH_3CN, and **7**·$CuBr_2$.

	**4**·$CuBr_4$	**5**·Cu_2Br_6	**6**·$CuBr_2$·CH_3CN	**7**·$CuBr_2$
CCDC number	1046219	1046220	1046221	1046222
Empirical formula	$C_{34}H_{28}O_4S_8CuBr_4$	$C_{34}H_{28}O_4S_8Cu_2Br_6$	$C_{40}H_{28}NO_8S_8CuBr_2$	$C_{42}H_{36}O_8S_8CuBr_2$
Formula weight	1140.22	1363.58	1130.48	1148.55
Temperature / K	293(2)	293(2)	293(2)	293(2)
λ / Å	0.71073	1.54184	0.71073	0.71073
Crystal size / mm^3	0.31×0.05×0.05	0.31×0.22×0.11	0.35×0.21×0.15	0.35×0.22×0.15
Crystal system	Orthorhombic	Triclinic	Triclinic	Triclinic
space group	*P*ccn	*P*-1	*P*-1	*P*-1
a / Å	12.9103(8)	8.8766(5)	8.0605(3)	12.8898(12)
b / Å	19.5008(5)	11.3774(5)	11.1170(7)	14.8147(13)
c / Å	16.3696(5)	12.1805(5)	13.7852(6)	14.8279(13)
α / °	90.00	92.375(3)	89.253(4)	107.327(8)
β / °	90.00	105.574(4)	88.684(4)	93.858(7)
γ / °	90.00	112.882(4)	80.707(4)	115.350(9)
V / $Å^3$	4121.2(3)	1076.89(9)	1218.68(10)	2379.7(3)
Z	4	1	1	2
d_{calc} / g·cm^{-3}	1.838	2.103	1.540	1.603
μ / mm^{-1}	4.850	11.681	2.478	2.538
$2\theta_{max}$ / °	57.1	145.28	57.26	57.18
Data / restraints / parameters	4816/0/231	4230/0/245	5499/0/286	10692/0/550
GooF	1.004	1.062	1.037	1.085
R [I>2σ(I)]	0.0516	0.0354	0.0601	0.0839
wR_2	0.0704	0.0899	0.1618	0.2016

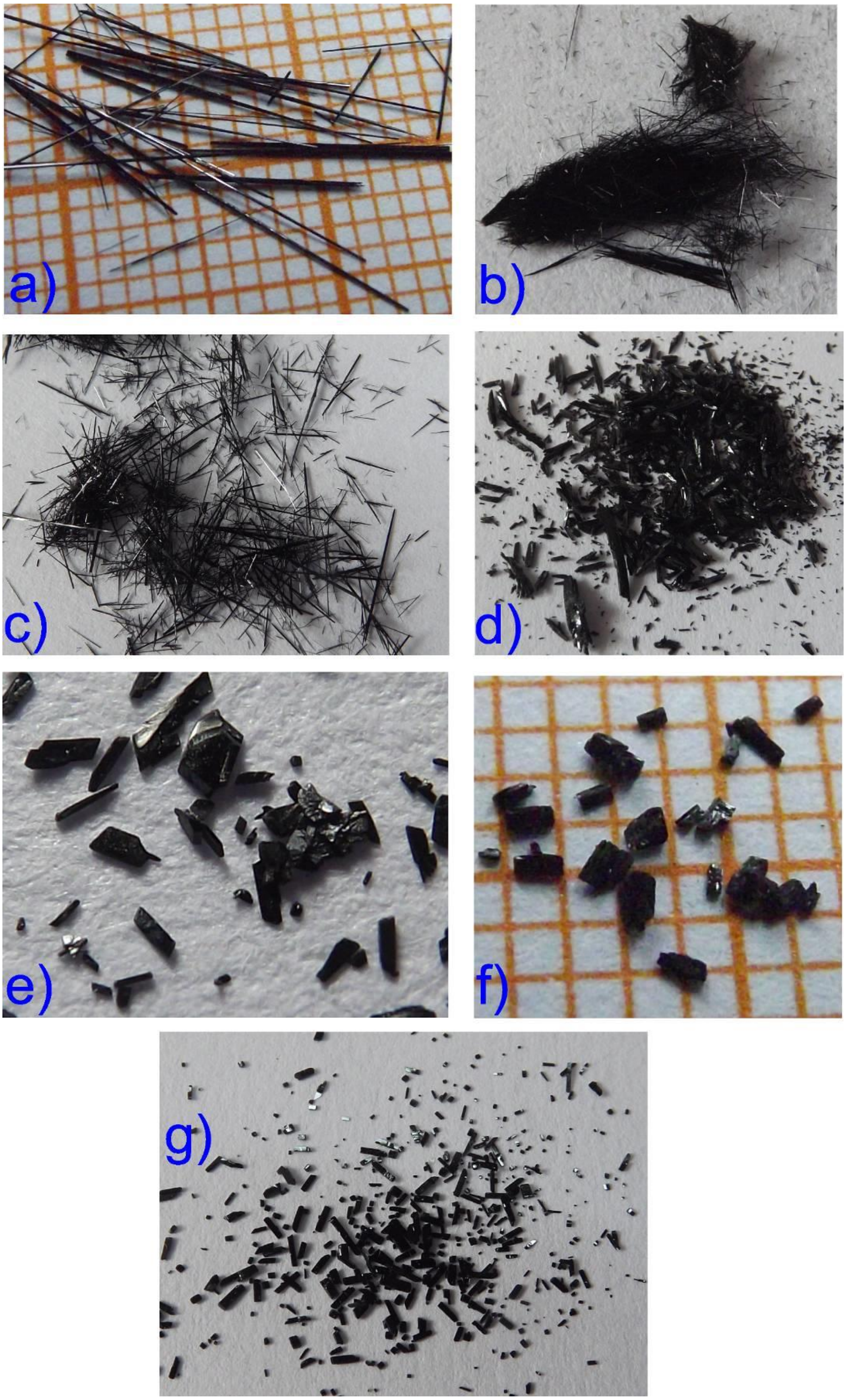

Figure S1: Photographs for the single crystals of salts: a) **1**·$CuBr_4$; b) **2**·$CuBr_4$; c) **3**·$(CuBr_4)_{0.5}$·$CuBr_3$·THF; d) **4**·$CuBr_4$; e) **5**·Cu_2Br_6; f) **6**·$CuBr_2$·CH_3CN; g) **7**·$CuBr_2$.

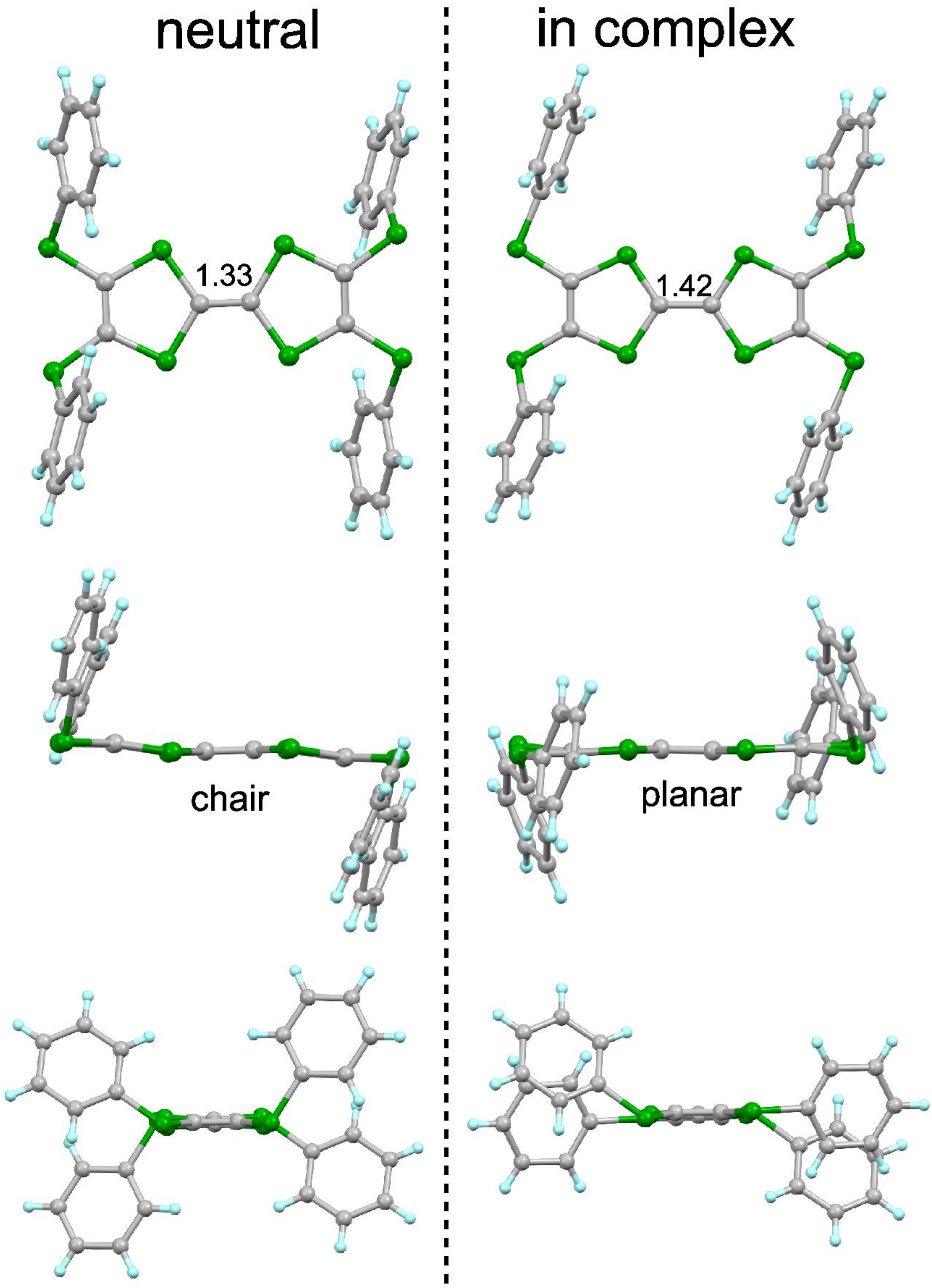

Figure S2: Comparison of the molecular geometry of **1** in the neutral state and in **1**·$CuBr_4$.

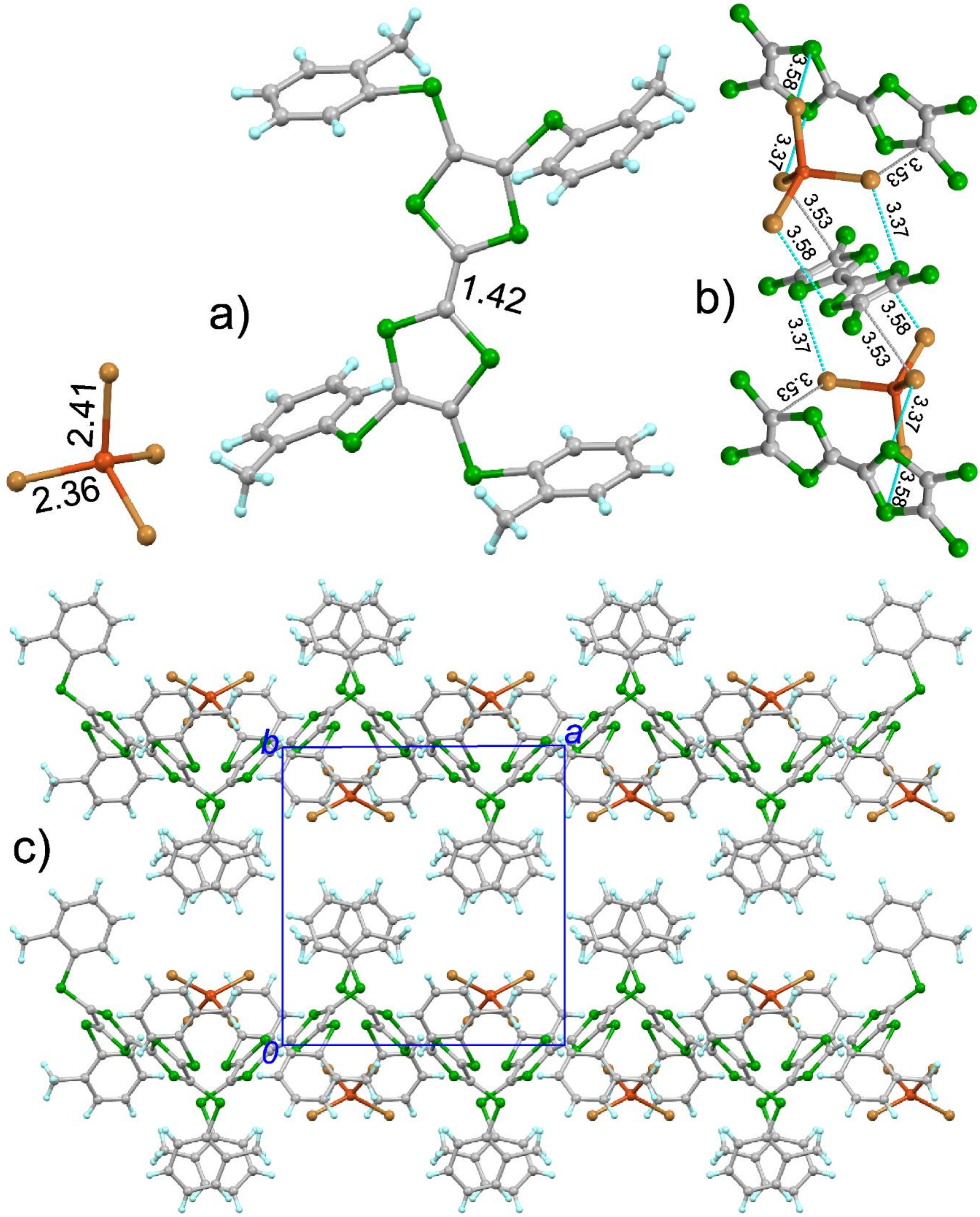

Figure S3: Crystal structure of **2**·$CuBr_4$: a) unit cell contents with the typical bond lengths shown (in unit of Å); b) interaction between $[Cu(II)Br_4]^{2-}$ ion and the central TTF core of **2**, where the cyan and grey dashed lines represent Br···S and Br···C contacts (Å), respectively; c) the packing structure viewed along the *a*-axis.

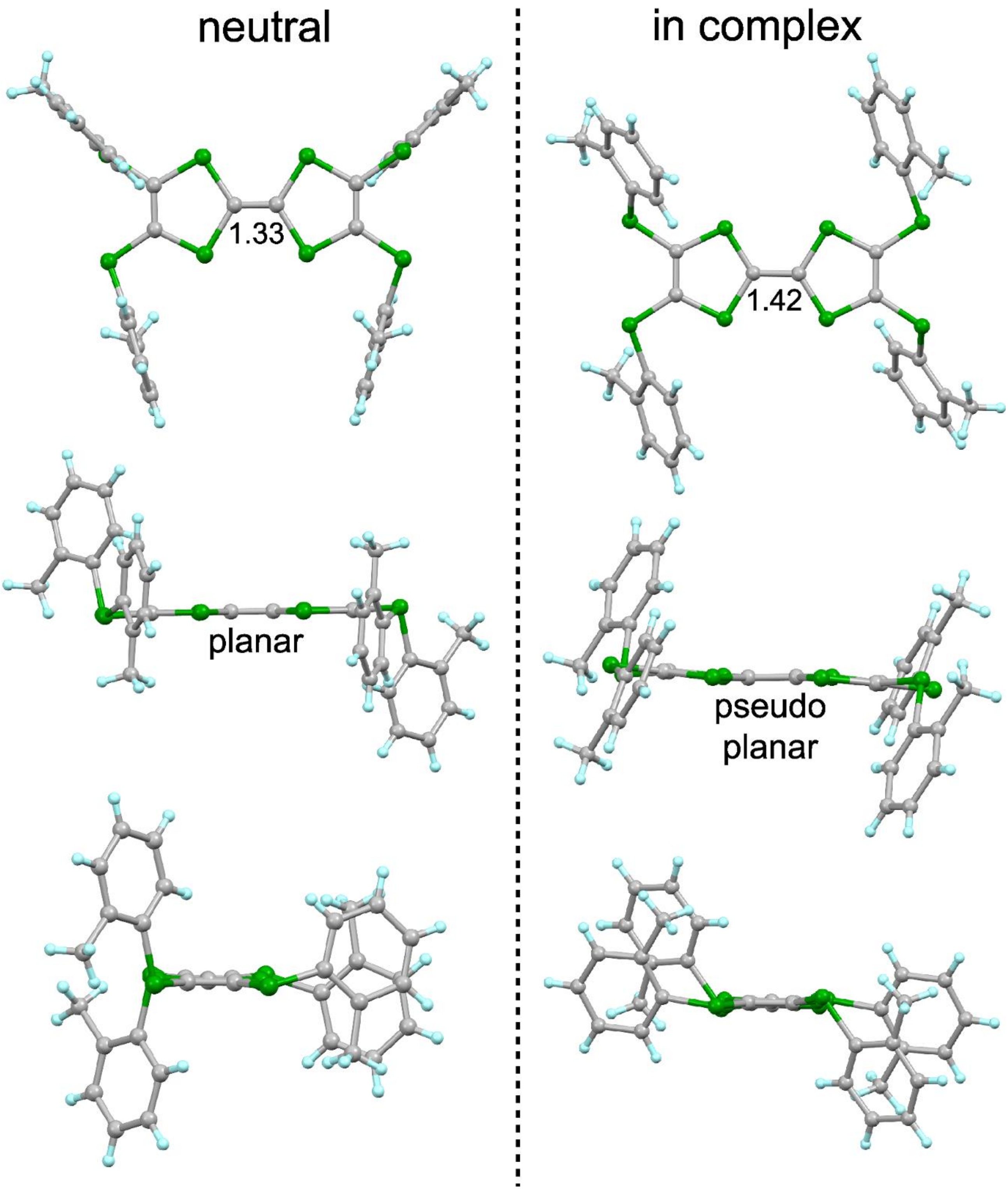

Figure S4: Comparison of the molecular geometry of **2** in the neutral state and in **2**·$CuBr_4$.

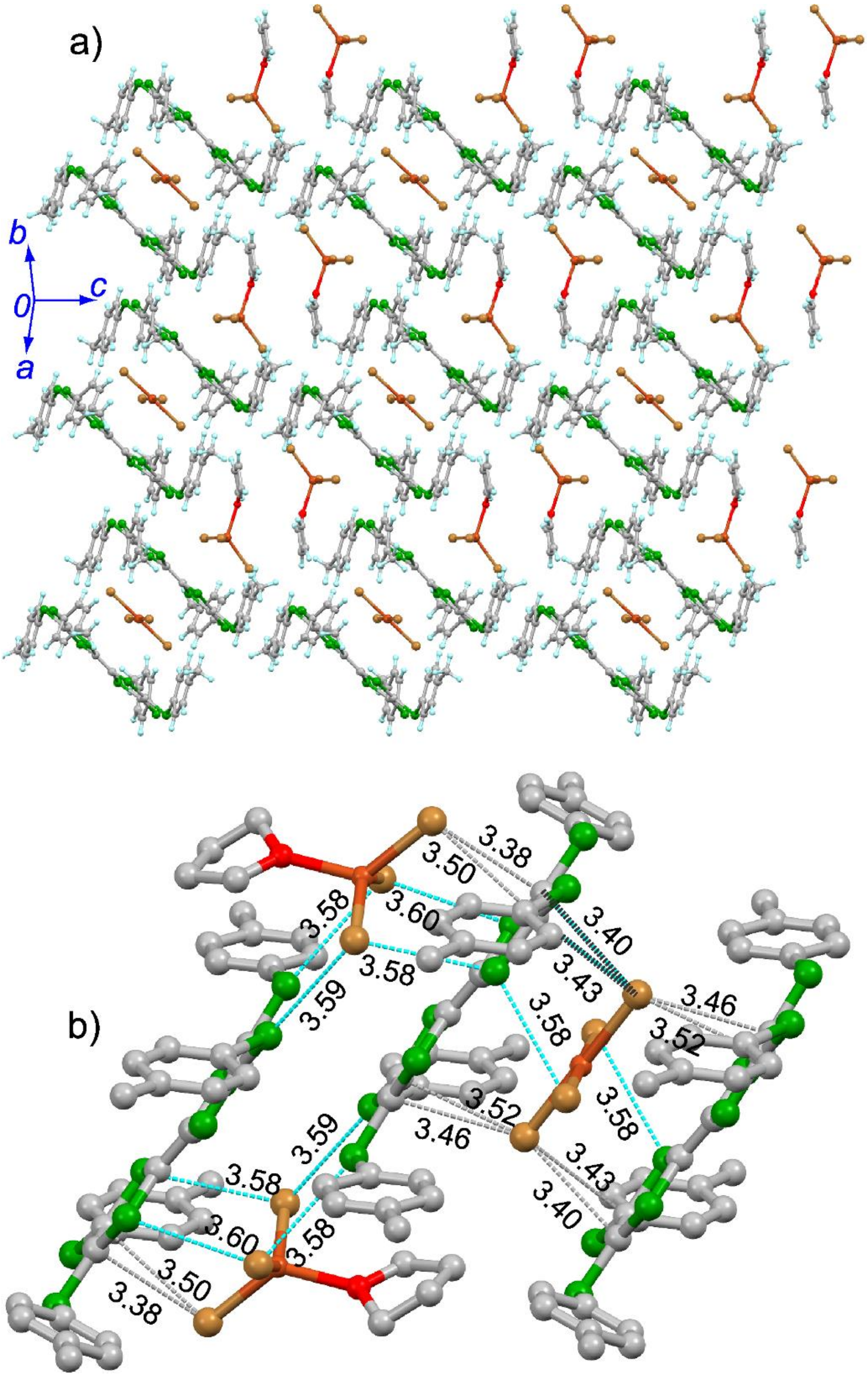

Figure S5: Crystal structure of **3**·$(CuBr_4)_{0.5}$·$CuBr_3$·THF at 173 K: a) packing structure; b) interaction between the anions and the central TTF core of **3**, where cyan and grey dashed lines represent Br···S and Br···C contacts (Å), respectively. In comparison with the structure at 291 K, the intermolecular interactions between **3** and anions at 173 K are significantly strengthened.

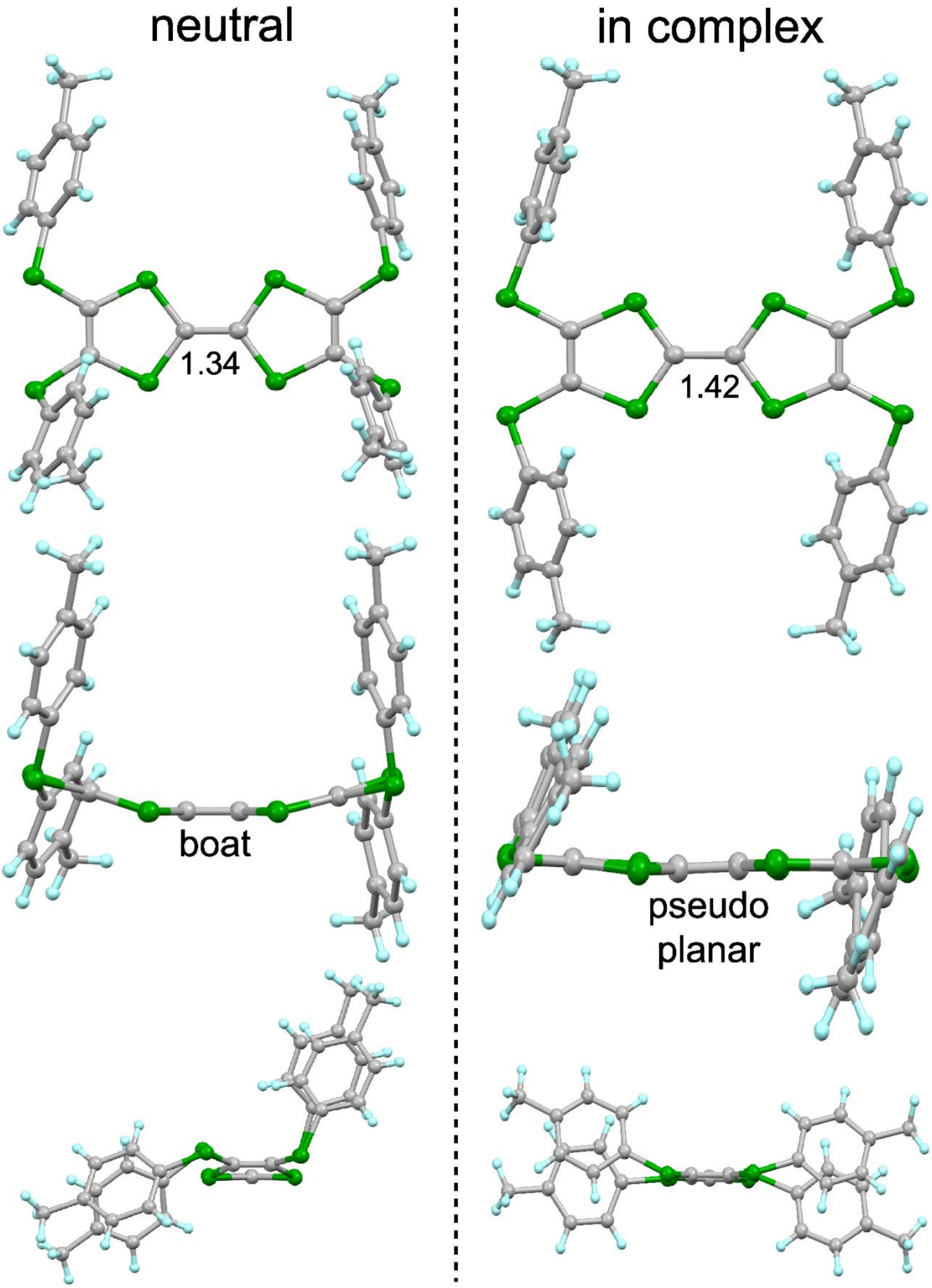

Figure S6: Comparison of the molecular geometry of **3** in neutral state and in **3**·$(CuBr_4)_{0.5}$·$CuBr_3$·THF.

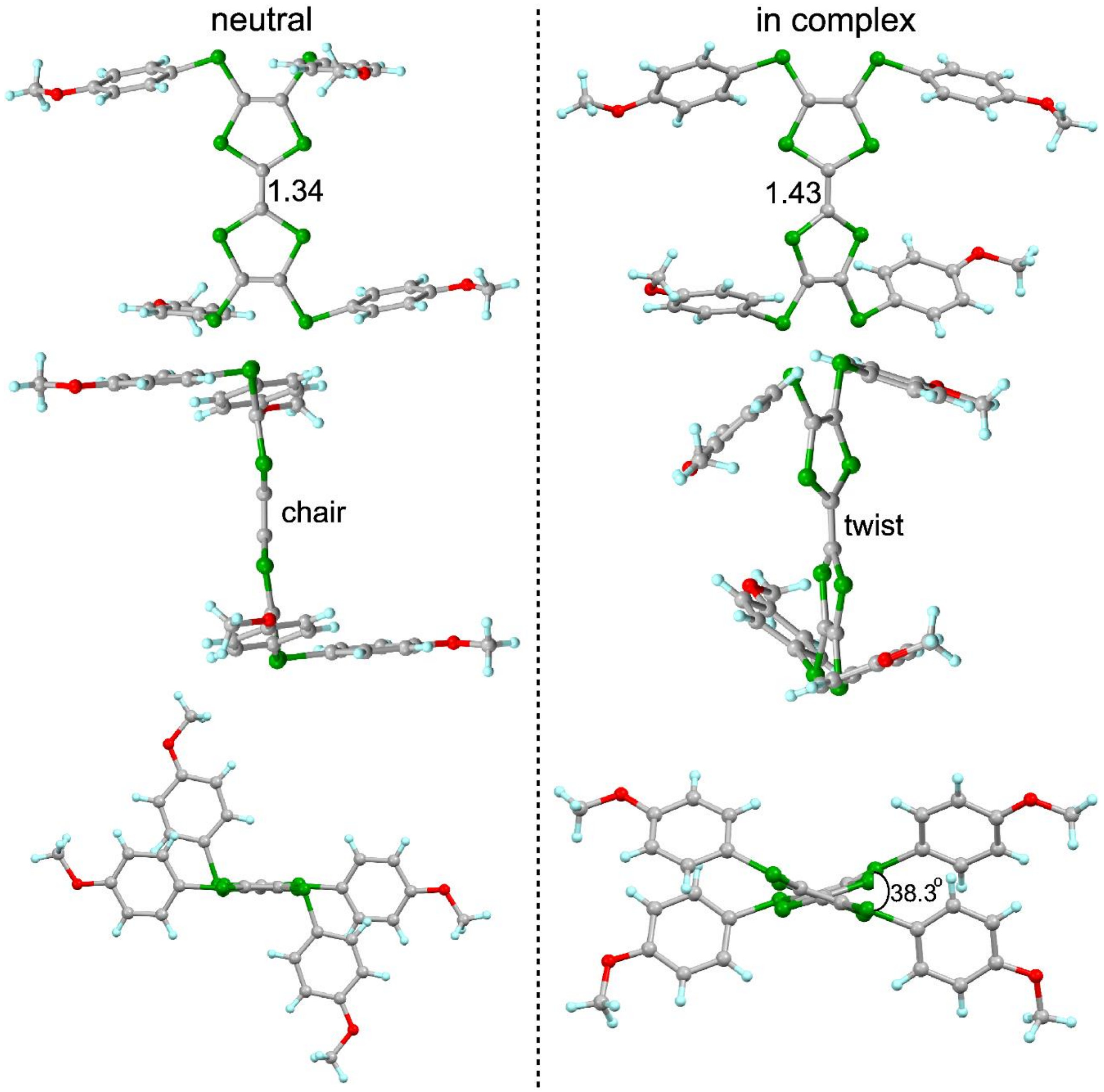

Figure S7: Comparison of the molecular geometry of **4** in the neutral state and in **4**·$CuBr_4$.

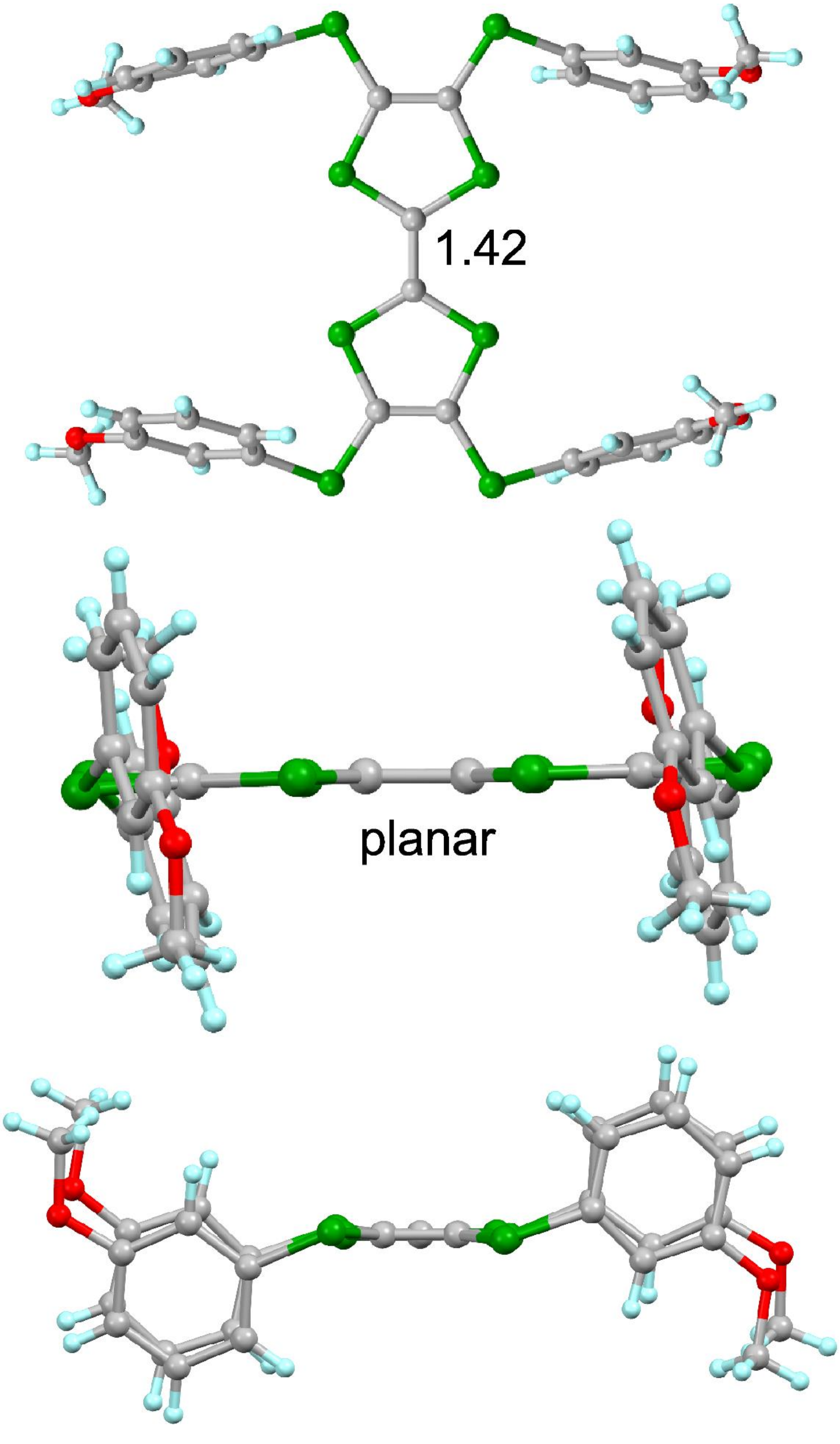

Figure S8: Molecular geometry of **5** in **5**·Cu_2Br_6.

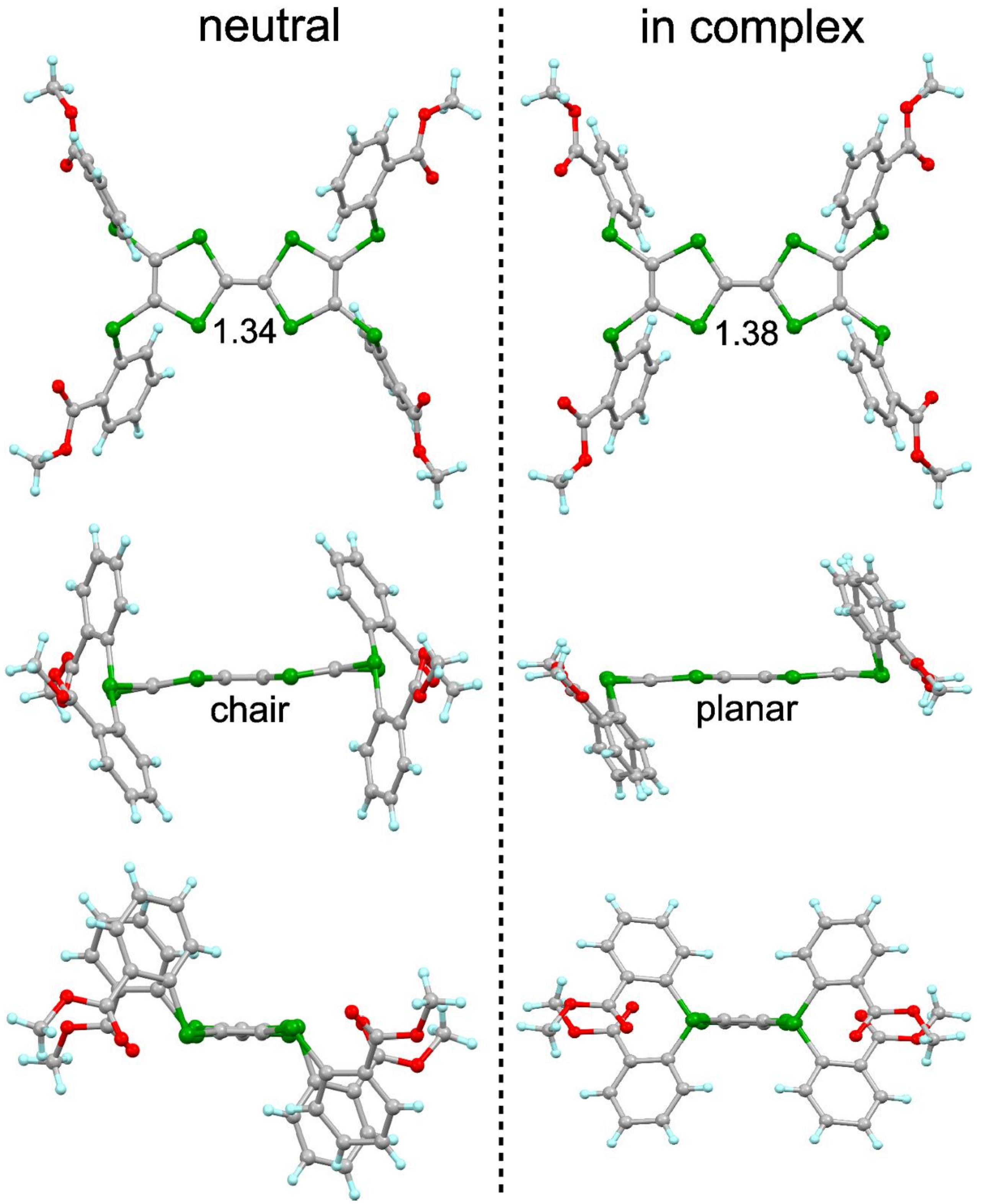

Figure S9: Comparison of the molecular geometry of **6** in neutral state and in **6**·$CuBr_2$·CH_3CN.

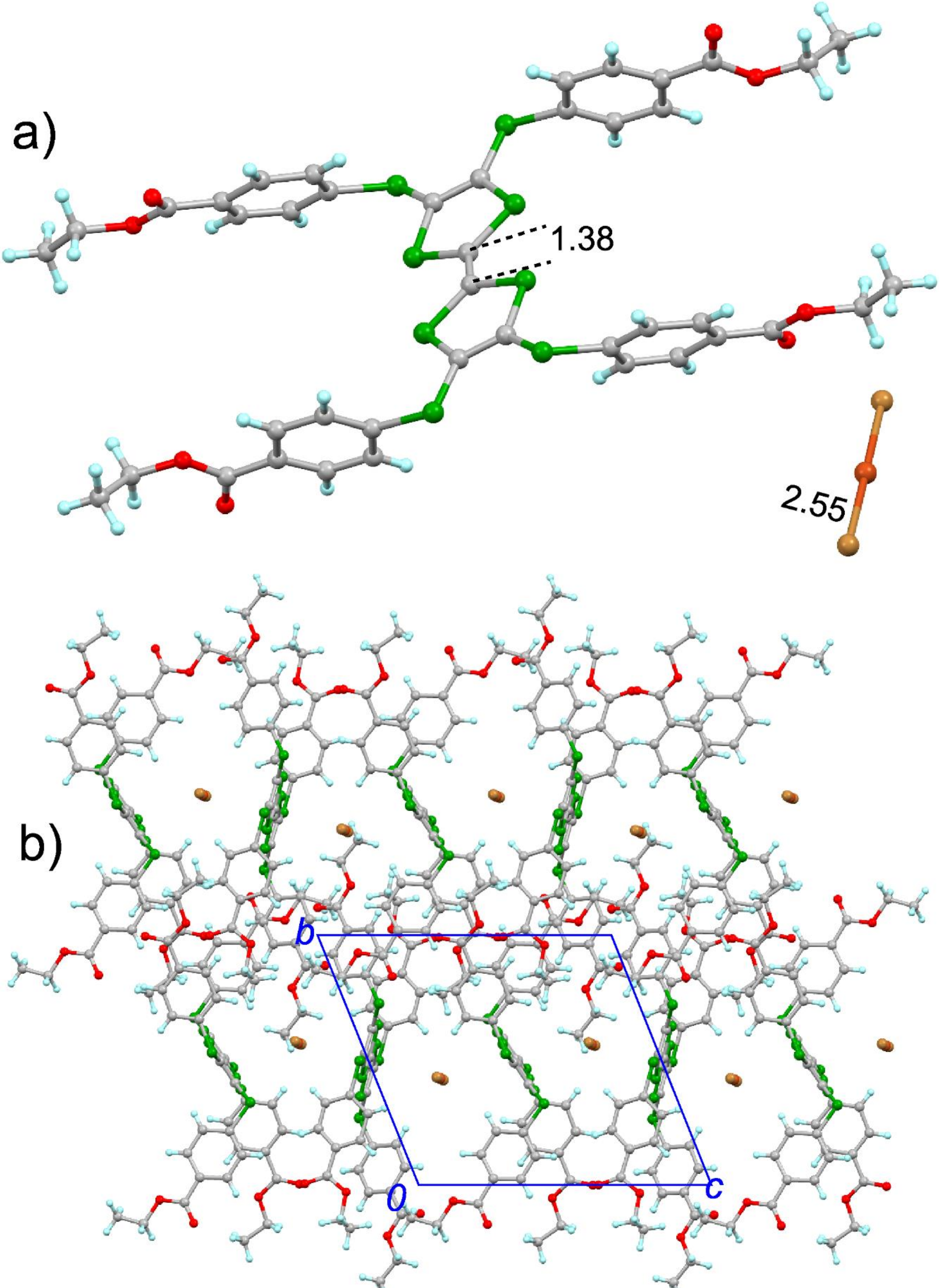

Figure S10: Crystal structure of **7**·$CuBr_2$: a) unit cell contents with the typical bond lengths shown (Å); b) packing structure viewed along the crystallographic *a*-axis.

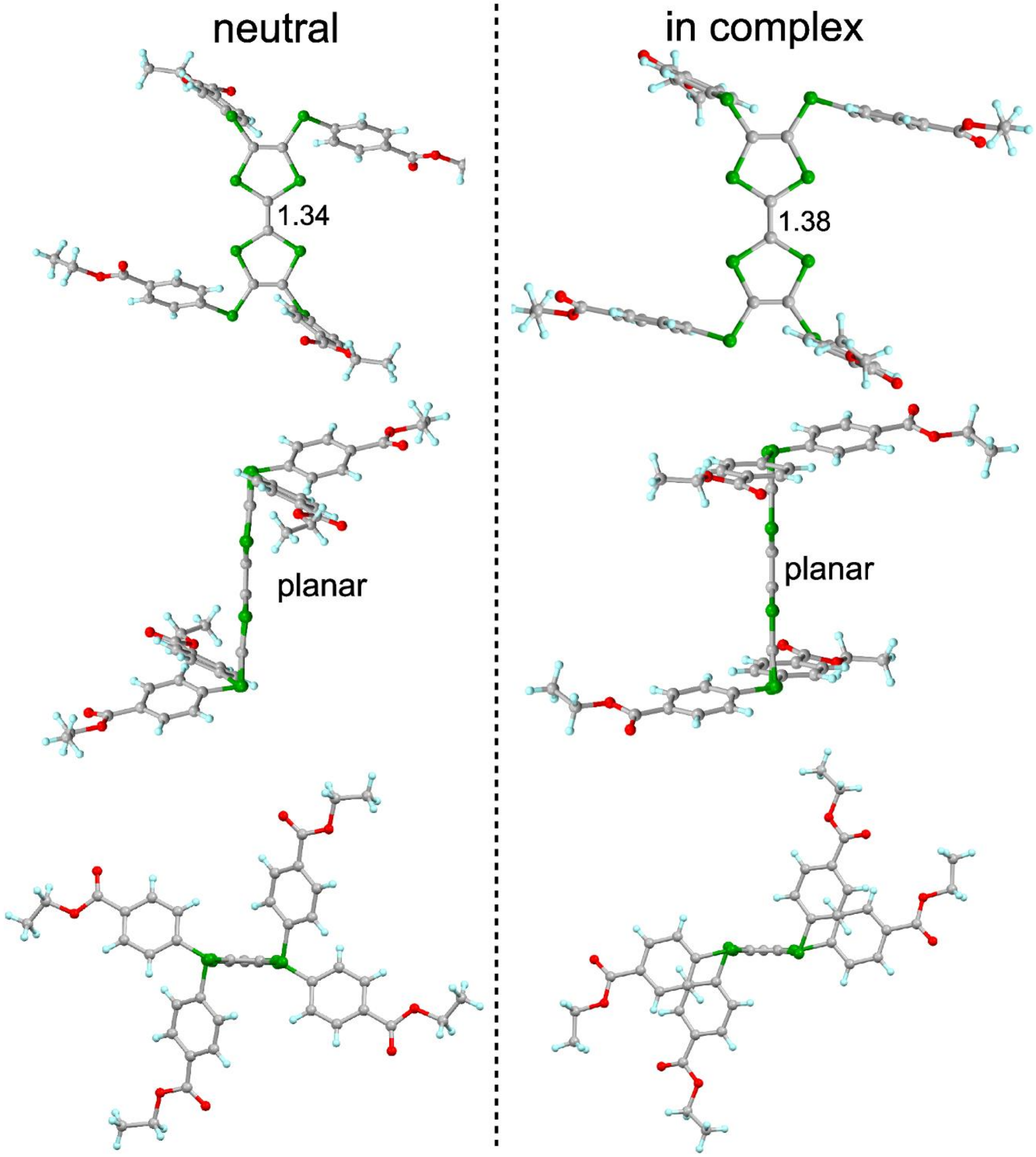

Figure S11: Comparison of the molecular geometry of **7** in the neutral state and in **7**·$CuBr_2$.
